# The Effects of Dipeptidyl Peptidase-4 Inhibitors on Cardiovascular Disease Risks in Type 2 Diabetes Mellitus

**DOI:** 10.1155/2013/459821

**Published:** 2013-04-22

**Authors:** Pegah Yousefzadeh, Xiangbing Wang

**Affiliations:** Division of Endocrinology, Metabolism and Nutrition, Department of Medicine, UMDNJ-Robert Wood Johnson Medical School, New Brunswick, NJ 08903, USA

## Abstract

*Objective*. To review the current literature investigating the effects of dipeptidyl peptidase-4 (DPP-4) inhibitors on the risk factors of cardiovascular disease (CVD). *Methods*. We conducted a search of PubMed and MEDLINE database, using the term DPP-4 inhibitor in combination with the following terms: metabolic syndrome, hypertension, dyslipidemia, insulin resistance, obesity, and CVD. We reviewed 100 relevant studies out of 227 articles, excluding single case reports, studies using animal models, and reports not written in English. We included 38 references in this review article. *Results*. The majority of the recent clinical studies have demonstrated that DPP-4 inhibitors have beneficial effects on cardiovascular (CV) system. These agents may have the potential to lower blood pressure, improve lipid profile and endothelial dysfunction, decrease the macrophage-mediated inflammatory response, and prevent myocardial injury. *Conclusion*. DPP-4 inhibitors have some CV protective effects in type 2 diabetes mellitus (T2DM) in addition to their antidiabetic actions. Long-term outcome clinical trials are under way to investigate the effects of the DPP-4 inhibitors on the elevated CV risks in patients with T2DM. Further investigation in a large cohort is warranted to assess the exact mechanisms of CV protective effects of DPP-4 inhibitors.

## 1. Introduction

T2DM is a well-studied chronic metabolic disease that when left untreated or insufficiently managed can cause serious microvascular and macrovascular complications. The blood glucose-lowering agents such as metformin, sulfonylurea derivatives, and insulin all can improve glycemic control in patients with T2DM, but these agents have limited or no effect on the associated CV risk factors accompanying T2DM including dyslipidemia, hypertension, and obesity. Treatment with both sulfonylurea derivatives and insulin has been associated with weight gain, which may diminish any positive effects on vascular endpoints, and thiazolidinediones have even been associated with an increased risk of CVD [1, 2]. Thus, there is a need to ensure that any new medication for T2DM is not associated with a deleterious effect on CV outcomes.

Glucose homeostasis is achieved by a complex interaction of hormones, principally insulin, glucagon, amylin, and incretins. Incretins are secreted from gastrointestinal tract in response to food intake and have several systemic effects, including glucose-dependent stimulation of insulin secretion by pancreatic beta-cells [3]. Two incretins have been identified: glucagon-like peptide-1 (GLP-1), derived from the L-cells of the distal small intestine and large bowel, and glucose-dependent insulinotropic polypeptide (GIP), derived from the K-cells of the proximal small intestine. Additional effects of GLP-1 include suppression of postprandial glucagon secretion from pancreatic alpha-cells, slowing of gastric emptying, and enhancement of satiety at a hypothalamic level leading to reduced food intake [4, 5]. GLP-1 and GIP are glucose-lowering agents that can interfere with postprandial hyperglycemia, which has been associated with CV complications [6]. G-protein-coupled receptors for GLP-1 are present in other tissues including cardiac myocytes, but their physiological action at these other sites remains unknown [7]. 

The enzyme dipeptidyl peptidase- (DPP-) 4, also known as adenosine deaminase complexing protein 2, degrades both GLP-1 and GIP to their inactive metabolites. Pharmacological competitive inhibition of DPP-4 increases the half-life and bioavailability of active incretins, enhancing their physiological effect. Currently available DPP-4 inhibitors include sitagliptin, saxagliptin, linagliptin, alogliptin, and vildagliptin. The first four are approved in the USA and throughout much of the world for the treatment of T2DM; vildagliptin has been approved for use in Europe and Latin America. Other members of this class are in phase III clinical trials and include dutogliptin and gemigliptin. With daily doses ranging from 100 mg for sitagliptin to 5 mg for saxagliptin and linagliptin, the drugs are all similar in their efficacy in lowering HbA1c levels, safety profiles, and patient tolerance [8]. DPP-4 inhibitors result in a mean decrease in A1C ranging between 0.5% and 1% [9]. In this review, we will focus on the protective CV effects of DPP-4 inhibitors. 

## 2. Methods

We conducted a search of PubMed and MEDLINE databases using the term DPP-4 inhibitor in combination with the following terms: metabolic syndrome, hypertension, dyslipidemia, insulin resistance, obesity, and CVD. We reviewed 100 relevant studies out of 227 articles. We excluded single case reports, studies using animal models and reports not written in English, and included 38 references in this review article.

## 3. Hypertension

T2DM and hypertension commonly coexist in the same patient. There have been several clinical studies of the potential effects of DPP-4 inhibitors on CV risk factors including hypertension. There are insufficient data to determine whether effects are directly associated with DPP-4 inhibition or mediated through the modulation of incretin hormone physiology. Shah et al. [10] have shown that inhibition of DPP-4 within the microcirculation relaxes vascular tone mediated by nitric oxide causing peripheral vasodilatation and decreased peripheral vascular resistance. The investigators proposed that the effect of this drug class on vascular relaxation might promote better control of blood pressure [11, 12]. Yet, in most DPP-4 trials in humans, no consistent effect on blood pressure has been demonstrated. 

In a single small study Mistry et al. [13] found that sitagliptin therapy in nondiabetic patients was associated with a 2-3 mmHg reduction in systolic blood pressure as assessed by 24-hour ambulatory monitoring. Conversely, Marney et al. [14] raised the possibility of an adverse haemodynamic interaction between DPP-4 inhibition and high-dose ACE inhibition in humans. When sitagliptin was used concurrently with enalapril, the antihypertensive effect of ACE inhibition was blunted. This effect was thought to be ensuing from activation of the sympathetic nervous system by substance P and decreased degradation of neuropeptide Y, resulting in downstream decreased vasodilatory effects. 

In a double-blind, randomized, multicenter, parallel group study, Bosi et al. [15] investigated treatment with 50 mg vildagliptin daily and 100 mg vildagliptin daily versus placebo in patients receiving metformin with inadequate glycemic control for 24 weeks. The results showed that both systolic and diastolic blood pressure tended to decrease during the study in each treatment group compared to placebo. The decrease in diastolic blood pressure in patients receiving 100 mg vildagliptin daily was significantly greater than that in patients receiving placebo. More studies will need to be done to elucidate the effects of DPP-4 inhibition on hypertension.

## 4. Dyslipidemia

Increased circulating levels of total cholesterol, low-density lipoprotein (LDL) cholesterol, high-density lipoprotein (HDL) cholesterol, and triglycerides are associated with elevated CVD risk in T2DM. In addition, nonfasting or postprandial hypertriglyceridemia has been independently associated with CVD risk [16]. In a large retrospective analysis from the General Electric Centricity database, patients with T2DM were treated with the DPP-4 inhibitor sitagliptin from January 1996 to January 2008. These patients showed decreases in LDL cholesterol, total cholesterol, and triglyceride levels [17]. Other studies in patients with T2DM treated with the DPP-4 inhibitors sitagliptin and vildagliptin reported decreases in levels of total cholesterol, LDL cholesterol, and triglycerides and an increase in HDL cholesterol [5]. Furthermore, the results from a meta-analysis, designed by Monami et al. [18] to assess the effect of DPP-4 inhibitors on lipid levels, showed that the difference in means for endpoint versus baseline total cholesterol in patients on DPP-4 inhibitors treatment was significantly higher in comparison with controls, meaning that treatment with DPP-4 inhibitors was associated with a significant reduction in total cholesterol.

In a randomized, double-blind, crossover study conducted by Boschmann et al. [19], twenty patients with T2DM and a body mass index between 28 and 40 kg/m^2^ underwent a 7-day treatment with the selective DPP-4 inhibitor vildagliptin or placebo. The authors concluded that the effects of DPP-4 inhibition on postprandial carbohydrate and lipid metabolism were tissue-specific. An increase in postprandial adipose tissue lipolysis was associated with an augmented postprandial systemic lipid oxidation rate. They speculated that the metabolic response to DPP-4 inhibition was due to sympathetic nervous system activation mediated through the GLP-1 receptor [19]. Matikainen et al. [20] assessed the effects of vildagliptin on postprandial lipid and lipoprotein metabolism in patients with T2DM, wherein treatment with the DPP-4 inhibitor improved triglyceride and apolipoprotein B-48 particle metabolism after a fat-rich meal. In general, no notable effects of the DPP-4 inhibition have been demonstrated on circulating lipid concentrations in most of clinical trials because lipid changes were not the primary outcome. 

## 5. Cardiovascular Disease

### 5.1. Cardiac Ischemic Events

Although it is evident that the CV protective effects of DPP-4 inhibition result from improving T2DM, a major risk factor for CV complications, accumulating evidence from experimental and clinical studies has suggested a direct effect of GLP-1 on the myocardium [21]. In vitro studies have shown that GLP-1 upregulates the expression of glucose transport protein- (GLUT-) 2 and 4, which in turn improves insulin resistance. GLUT-4 expression is markedly reduced in T2DM. GLP-1 mediates GLUT-4 translocation to the cardiac myocyte surface to increase glucose uptake [22].

 In a recent study, a post hoc assessment of CV safety in patients with T2DM was performed by pooling data from 25 double-blind studies, which randomized patients at baseline to sitagliptin or a nonsitagliptin comparator. Included studies were limited to those ranging from 12 to 104 weeks in duration. Major adverse cardiovascular events (MACEs) including ischemic events and CV deaths were analyzed. In the entire cohort analysis, 78 patients had at least 1 reported MACE-related event, with 40 in the sitagliptin group and 38 in the nonexposed group. In this analysis, the exposure-adjusted incidence rate was 0.65 per 100 patient-years in the sitagliptin group and 0.74 in the nonexposed group; comparing sitagliptin to placebo, the exposure-adjusted incidence rate was 0.80 per 100 patient-years with sitagliptin and 0.76 with placebo; comparing sitagliptin to sulfonylurea, the exposure-adjusted incidence rate was 0.00 per 100 patient-years with sitagliptin and 0.86 with sulfonylurea. In conclusion, a pooled analysis of 25 randomized clinical trials did not indicate that treatment with sitagliptin increases CV risk in patients with T2DM. In a subanalysis, a higher rate of CV-related events was associated with sulfonylurea relative to sitagliptin [23].

In a meta-analysis conducted by Patil et al. [24], DPP-4 treatment as compared to other oral hypoglycemic agents was associated with reduced CV events. The results of this meta-analysis showed that the overall use of DPP-4 inhibitors was associated with a lower risk of adverse CV effects and a lower risk of nonfatal myocardial infarction (MI) or acute coronary syndrome (ACS) compared to placebo or other oral hypoglycemic agents. Subgroup analysis by the studied DPP-4 inhibitors showed a significantly lower risk of adverse CV events with sitagliptin, but not with saxagliptin, alogliptin, or vildagliptin. Risk of adverse CV events with DPP-4 inhibitor therapy was not significantly different compared to placebo but was significantly lower compared to metformin and other oral hypoglycemic agents including sulfonylureas and thiazolidinediones. In addition, cardioprotective effects have been noted with the DPP-4 inhibitor sitagliptin in other clinical studies. In one study, 14 patients with coronary artery disease and preserved left ventricular function awaiting revascularization were studied. After a single dose of sitagliptin or placebo, glucose 75 mg was given orally to promote GLP-1 secretion, and dobutamine stress echocardiography was conducted with tissue doppler imaging at rest, peak stress, and 30 minutes later. Sitagliptin increased plasma GLP-1 levels at peak stress from 10.0 ± 9.0 to 17.0 ± 11.0 pg/mL (*P* ≤ 0.003) and in recovery from 9.0 ± 6.0 to 12.0 ± 6.0 pg/mL, and ejection fraction from 64.0 ± 8.0% to 73.0 ± 7.0% [25].

In a large meta-analysis, conducted by Schweizer et al. [26], data were pooled from 25 phase III studies of vildagliptin, used either as a monotherapy or combination therapy, with durations of 12 weeks to 2 years. The safety of vildagliptin was assessed relative to a pool of both placebo and active comparators. CV and cerebrovascular (CCV) events were adjudicated in a prospective, blinded fashion by an independent CCV adjudication committee. Meta-analysis of confirmed CCV events was performed. Categories included in the composite endpoint were ACS, transient ischemic attack with imaging evidence of infarction, stroke, and CCV death. Subgroup analyses by age, gender, and CV risk status were also carried out. Relative to all comparators, the RRs for the composite endpoint were <1 for both vildagliptin 50 mg qd and vildagliptin 50 mg bid. The results were consistent across subgroups defined by age, gender, and CV risk status, including the higher CV risk subgroups of elderly patients, males, or patients with a high CV risk status. The exposure-adjusted incidences of each component of the composite endpoint for vildagliptin 50 mg bid were also lower than or similar to those of all comparators. They concluded that vildagliptin was not associated with an increased risk of adjudicated CCV events relative to all comparators in the broad population of T2DM including patients at increased risk of CCV events.

Monami et al. [27] conducted a meta-analysis of randomized clinical trials to assess the effect of DPP-4 inhibitors on the incidence of MACE, cancer, and pancreatitis. Fifty-three trials enrolling 20,312 and 13,569 patients for DPP-4 inhibitors and comparators, respectively, were included, reporting 176 malignancies, 257 MACEs, and 22 pancreatitis. DPP-4 inhibitors, compared with placebo or other treatment, were associated with a similar risk of cancer and pancreatitis, and with a reduced risk of MACE. In conclusion, this meta-analysis seemed to exclude any relevant short-term effect of DPP4-inhibitors on the incidence of cancer and suggested a possible protection from MACE, but the result should be interpreted with caution, as those events were not the principal endpoints.

Frederich et al. [28] sought to assess the relative risk of CV events across eight randomized double-blinded phase II and III trials evaluating saxagliptin in patients with T2DM. Data were pooled from randomized patients treated with saxagliptin, placebo, metformin, or glyburide. CV events were defined as death, MI, stroke, revascularization procedures, and cardiac ischemia. Cox proportional regression hazard model pointed to a 41% RR reduction of CV events with saxagliptin as compared with the comparators. The composite of CV death, MI, or stroke was confirmed in 40 patients, with 22 exposed to saxagliptin (0.7%) and 18 to comparator (1.4%). The Cox RR estimate was 0.43, translating to a 57% risk reduction in patients assigned to saxagliptin therapy. The authors concluded that their data suggested no CV harm and a potential for an actual reduction in CV events with saxagliptin. The study is limited due to its retrospective nature and small number of classifiable events; moreover, the trials were not initially designed to assess CV safety, so the results must be considered highly preliminary [28].

### 5.2. Markers of Inflammation

Monocyte chemoattractant protein-1 (MCP-1) is a proinflammatory cytokine that has been shown to be higher in patients with atherosclerosis and during MI and ischemic stroke. Stromal cell-derived factor- (SDF-) 1*α* is a chemoattractant for human CD34-positive cells which may have a role in myocardial regeneration. Also, endothelial progenitor cells (EPCs) are a subset of bone marrow-derived cells that have a role in the maintenance and preservation of vascular turnover, remodeling, and homeostasis [29]. In a controlled, nonrandomized clinical trial, EPC levels, MCP-1, and plasma concentrations of SDF-1*α* were measured in T2DM patients who had been treated with sitagliptin for 4 weeks and in 16 patients who had not received additional treatment on top of metformin and/or sulfonylurea derivatives. Sitagliptin therapy increased EPC levels and led to the upregulation of SDF-1*α*. The proinflammatory chemokine MCP-1 was decreased in the sitagliptin-treated patients. Functional EPCs represent a prerequisite for a healthy CV system in diabetic patients, and this ancillary effect of DPP-4 inhibition might have potential favorable CV implications [30, 31].

### 5.3. Other Cardiovascular Effects

Patients with T2DM suffer from obesity-related conditions induced by insulin resistance. Endothelial dysfunction has been recognized as an early phenomenon in diabetic vascular disease and may be one of the key conditions contributing to further CV morbidity in patients with T2DM. Postprandial hyperglycemia and postprandial hypertriglyceridemia are associated with oxidative stress, endothelial dysfunction, decreased fibrinolysis, sympathetic activation, and increased atherosclerotic coronary plaque burden [32]. A recent study by Matsubara et al. [33] assessed changes in endothelial function in 40 Japanese patients with coronary artery disease and diabetes, treated with or without sitagliptin for 6 months. Starting mean HbA1c was 7.4%. Patients were assigned (not randomized) to sitagliptin or conventional therapy. Endothelial function was assessed by reactive hyperemia peripheral arterial tonometry index (RHI). Systolic blood pressure was significantly lower by 7 mmHg in the sitagliptin group, whereas systolic BP rose by 3 mm in the nonsitagliptin group, representing a final treatment difference of 13 mmHg between groups. HbA1c was not different after 6 months. Patients with sitagliptin experienced a greater improvement in RHI (endothelial function) relative to the control group, and reductions in C-reactive protein correlated with improved endothelial function. The authors concluded that sitagliptin significantly improved endothelial function and inflammatory state in patients with CAD and uncontrolled T2DM, beyond its hypoglycemic action.

## 6. Clinical Studies

At present, there are large ongoing multicenter clinical trials testing the cardioprotective effects of DPP-4 inhibitors, which, when completed, may provide the needed confirmatory evidence for their cardioprotective effects. EXAMINE (Cadiovascular Outcomes Study of Alogliptin in Subjects with T2DM and ACS) is an ongoing multicenter randomized double-blinded, placebo-controlled superiority trial evaluating CV outcomes following treatment with alogliptin in addition to standard care in subjects with T2DM and recent ACS. The study group will be compared with standard therapy without DPP-4 inhibition. The primary outcome measure will be time from randomization to the first occurrence of a primary cardiac event, defined as a composite of CV death, nonfatal MI, and nonfatal stroke. This 5400-patient study completed recruitment in December 2013 [34].

CAROLINA (Cardiovascular Outcome Study of Linagliptin versus Glimepiride in Patients with T2DM) is a long-term multicenter study that is currently enrolling and plans to enroll 6,000 patients with a completion date in September 2018 [35]. SAVOR-TIMI (Does Saxagliptin Reduce the Risk of Cardiovascular Events When Used Alone or Added to Other Diabetes Medications?) is an interventional long-term multicenter trial that is currently enrolling and plans to enroll 16,550 patients with a completion date in April 2014 [36]. TECOS (Randomized, Placebo-Controlled Clinical Trial to Evaluate Cardiovascular Outcomes after Treatment with Sitagliptin in Patients With T2DM and Inadequate Glycaemic Control) is a phase III noninferiority trial designed to assess CV outcomes of long-term treatment with sitagliptin in patients with T2DM (HbA1c of 6.5–8.0%) and a history of CVD. The study group will be compared with those patients treated with usual standard of care. The primary outcome measure will be time to first confirmed CV event, a composite of CV-related death, nonfatal MI, nonfatal stroke, or unstable angina requiring hospitalization. Fourteen thousand participants are estimated to be recruited into TECOS, with an estimated study completion date of December 2014 [37]. Also, the interim analysis of results of SITAGRAMI (Safety and Efficacy of Sitagliptin plus Granulocyte Colony-Stimulating Factor in Patients Suffering from Acute Myocardial Infarction), a phase III multicenter trial testing the myocardial regenerating effects after an acute MI of the combination of sitagliptin with G-CSF, are encouraging but need to be confirmed with completion of the long-term study [38].

## 7. Conclusion

DPP-4 inhibitors have some CV protective effects in T2DM in addition to their antidiabetic actions. Additional benefits include lowering the blood pressure, improving the lipid profile and the endothelial dysfunction, decreasing the macrophage-mediated inflammatory response, and reducing myocardial injury. Further investigation in a large cohort is warranted to assess the exact mechanisms of CV protective effects of DPP-4 inhibitors.
